# Prognostic Prediction Models Based on Clinicopathological Indices in Patients With Resectable Lung Cancer

**DOI:** 10.3389/fonc.2020.571169

**Published:** 2020-10-29

**Authors:** Yanyan Liu, Xinying Li, Zhucheng Yin, Ping Lu, Yifei Ma, Jindan Kai, Bo Luo, Shaozhong Wei, Xinjun Liang

**Affiliations:** ^1^ Division of Internal Medicine, Department of Nephrology, Tongji Hospital, Tongji Medical College, Huazhong University of Science and Technology, Wuhan, China; ^2^ Department of Epidemiology and Biostatistics, The Ministry of Education Key Lab of Environment and Health, School of Public Health, Huazhong University of Science and Technology, Wuhan, China; ^3^ Department of Medical Oncology, Hubei Cancer Hospital, Tongji Medical College, Huazhong University of Science and Technology, Wuhan, China; ^4^ Department of Gastrointestinal Surgery, Hubei Cancer Hospital, Tongji Medical College, Huazhong University of Science and Technology, Wuhan, China; ^5^ Department of Thoracic Surgery, Hubei Cancer Hospital, Tongji Medical College, Huazhong University of Science and Technology, Wuhan, China

**Keywords:** lung cancer, prognosis, time-dependent receiver operating characteristic curve, nomogram, clinicopathological indices

## Abstract

Serum enzymes, blood cytology indices, and pathological features are associated with the prognosis of patients with lung cancer, and we construct prognostic prediction models based on clinicopathological indices in patients with resectable lung cancer. The study includes 420 patients with primary lung cancer who underwent pneumonectomy. Cox proportional hazards regression was conducted to analyze the prognostic values of individual clinicopathological indices. The prediction accuracies of models for overall survival (OS) and progression-free survival (PFS) were estimated through Harrell’s concordance indices (C-index) and Brier scores. Nomograms of the prognostic models were plotted for individualized evaluations of death and cancer progression. We find that the prognostic model based on alkaline phosphatase (ALP), lactate dehydrogenase (LDH), age, history of tuberculosis, and pathological stage present exceptional performance for OS prediction [C-index: 0.74 (95% CI, 0.69-0.79) and Brier score: 0.10], and the prognostic model based on ALP, LDH, and platelet distribution width (PDW), age, pathological stage, and histological type presented outstanding performance for PFS prediction [C-index: 0.71 (95% CI, 0.66-0.75) and Brier score: 0.18]. These findings show that the models based on clinicopathological indices might serve as economic and efficient prognostic tools for resectable lung cancer.

## Introduction

Lung cancer is the leading cause of cancer morbidity and mortality worldwide. An estimated 2,093,876 new cases (11.6% of all sites) and 1,761,007 deaths (18.4% of all sites) of lung cancer occurred in 2018 (1). It is conservatively estimated that 35.8% of patients with non-small cell lung cancer) develop locally advanced disease or metastases (2). Although new therapies, such as molecularly targeted therapy and immunotherapy, have provided a greater chance to improve the prognosis of patients with lung cancer (3), the overall 5-year survival rate of lung cancer is no more than 20% (4).

Traditional prognostic prediction for cancer mainly relies on pathological features, such as tumor node metastasis (TNM) stage. However, even at the same stage, the clinical processes of patients with cancer are not exactly the same (5). Subtype lung cancer is associated with survival, small-cell lung cancer is characterized by a high growth fraction and early development of widespread metastases (6), and the 5-year survival rate is lower than 10% (7). Smoking increases the risk of lung cancer incidence and poor survival, and the implementation of tobacco control efforts could reduce lung cancer rates (8). In addition to these clinicopathological features, some routine clinical indices, including serum enzymes and blood cytology indices, are proven to be associated with the prognosis of lung cancer, including the prognostic nutritional index (PNI) (9), albumin-to–alkaline phosphatase ratio (10), neutrophil-to-lymphocyte ratio (NLR) (11, 12), lactate dehydrogenase (LDH) (13) and alkaline phosphatase (ALP) (14). However, the performance of the combination of these clinicopathological indices in the prognostic prediction of lung cancer is still unknown.

Therefore, this study was designed to identify the values of serum enzymes and blood cytology indices in lung cancer prognosis and to develop prognostic models based on serum enzymes, blood cytology indices, and pathological features to improve the accuracy of survival prediction in patients with lung cancer.

## Methods

### Patients and Follow-up Methods

Four hundred eighty-seven patients with primary lung cancer who underwent first tumor resection at Hubei Cancer Hospital from January 2015 to June 2017 were recruited. The patients were excluded if (1) they censored within 90 days from tumor resection; (2) they had previous or concurrent malignancies; (3) they had preexisting inflammatory conditions, such as active or chronic infection. Finally, 420 patients were included in this study. Written informed consent was obtained from all patients before this study. The study was approved by the ethical committee of Hubei Cancer Hospital.

We obtained outcomes by reviewing medical records and making follow-up calls. The main outcome was overall survival (OS), and the secondary outcome was progression-free survival (PFS). OS was defined as the interval from the date of tumor resection to the date on which the patient died from any cause, was lost to follow-up, or the end of the follow-up, whichever came first. PFS was defined as the interval from the date of tumor removal to the date on which a patient died, recurrence or metastasis was detected, loss to follow-up, or the end of the follow-up, whichever came first. Follow-up was carried out until the end of February 2019.

### Data Collection

The preoperative clinicopathological characteristics of all participants were extracted from the hospital’s medical records. The serum enzymes and blood cytology indices included alanine aminotransferase (ALT), aspartate aminotransferase (AST), ALP, LDH, albumin, neutrophil, monocyte, lymphocyte, platelet, and platelet distribution width (PDW). Tuberculosis (TB) evaluation: (1) the history of TB; (2) the patients included in the study underwent chest computed tomography scans, so the radiologic signs (such as punctuate calcification and cord high-density shadows) of old TB could be found. We combine these two criteria to determine the presence of TB, and all patients are unified. Other clinicopathological characteristics include age, sex, smoking status, height, weight, history of TB, pathological stage, histological type, number of tumor invading lung lobes, tumor size, and neoadjuvant therapy.

### Statistical Analysis

Continuous variables were presented as the mean ± standard deviation or median and interquartile range, and Student’s *t* test or the Wilcoxon test was used for comparisons between groups. Categorical variables were expressed by counts and percentages, and the chi-square test was used for comparison between groups. Five joint indices were constructed, including the NLR, MLR, platelet-to-lymphocyte ratio (PLR), PNI, and systemic inflammation index (SII). Receiver operating characteristic (ROC) curves were applied to transform the continuous variables (ALT, AST, ALP, LDH, PDW, NLR, MLR, PLR, PNI, and SII) into dichotomized variables by using inflexion points as cutoffs. Kaplan-Meier survival curves and log-rank tests were used to compare the survival differences between groups classified by dichotomized clinicopathological indices. Univariate and multivariate Cox proportional hazards regression were applied to detect the associations of individual clinicopathological features, and integrated predictive model-based serum enzymes, blood cytology indices, and other clinicopathological characteristics with OS/PFS by calculating hazard ratios (HRs) and 95% confidence intervals (95% CIs). The prognostic efficacy of the predictive models was estimated by Harrell’s concordance index (C-index) and the Brier score. Time-dependent ROC curves and calibration curves were plotted to visualize the performance of the models (15). Nomograms of the predictive models were plotted for individualized evaluation of OS and PFS.

All statistical tests were two-sided, and *P<0.05* was considered statistically significant. The time-dependent ROC curve, calibration curve, and nomogram were performed using the “survival ROC” “time ROC” “pec” and “regplot” packages of R 3.6.0 (The R Foundation for Statistical Computing, Vienna, Austria). Other statistical analyses were performed using SAS Statistics software 9.4 (SAS Institute Inc., Cary, North Carolina, USA).

## Results

### Characteristics of Study Patients

A total of 420 patients were included in this study, including 282 (67.14%) males and 138 (32.86%) females. The baseline characteristics of the patients are shown in **Table 1**. Among the patients included in the study, 48.10% were previous or current smokers, and 6.67% had a history of tuberculosis. Tumors of stages I, II, III, and IV accounted for 43.81%, 24.52%, 25.48%, and 6.19%, respectively. A total of 243 patients had lung adenocarcinoma, accounting for 55.33%. The median follow-up time was 31.82 months. At the end of follow-up, 80 (19.05%) patients had died and 142 (33.81%) presented with cancer progression. The dead cases were more likely to be males, smokers, to have advanced pathological stage, and to have received neoadjuvant therapy. Similarly, the cases presenting tumor progression were more likely to have advanced pathological stage and a larger number of tumor-invading lung lobes. Based on Cox regression analyses, advanced pathological stage and history of TB were independent risk factors for OS, and advanced pathological stage and histological type were independent risk factors for PFS (**Tables S1, S2**).

**Table 1:** The characteristics of patients with lung cancer.

Variables		Survival		*P^a^*	Cancer progression^b^		*P^a^*
		Alive (n=340)	Dead (n=80)		No (n=278)	Yes (n=142)	
Age at diagnosis, y	Median (IR)	60.00 (11.00)	62.00 (9.50)	0.24	60.00 (11.00)	61.00 (9.00)	0.42
Sex (n, %)	Male	220 (64.71)	62 (77.50)	0.03	179 (64.39)	103 (72.54)	0.09
	Female	120 (35.29)	18 (22.50)		99 (35.61)	39 (27.46)	
Smoking (n, %)	No	189 (55.59)	29 (36.25)	0.02	152 (54.68)	66 (46.48)	0.10
	Yes	151 (44.41)	51 (63.75)		126 (45.32)	76 (53.52)	
Body mass index, kg/m^2^	Median (IR)	23.23 (4.09)	22.66 (3.96)	0.29	23.24 (4.04)	22.69 (4.28)	0.22
History of tuberculosis (n, %)	No	321 (94.41)	71 (88.75)	0.07	262 (94.24)	130 (91.55)	0.29
	Yes	19 (5.59)	9 (11.25)		16 (5.76)	12 (8.45)	
Pathological stage (n, %)	Stage I	169 (49.71)	15 (18.75)	<0.0001	156 (56.12)	28 (19.72)	<0.0001
	Stage II	88 (25.88)	15 (18.75)		65 (23.38)	38 (26.76)	
	Stage III	66 (19.41)	41 (51.25)		46 (16.55)	61 (42.96)	
	Stage IV	17 (5.00)	9 (11.25)		11 (3.96)	15 (10.56)	
Histological type (n, %)	Adenocarcinoma	202 (59.41)	41 (51.25)	0.12	166 (59.71)	77 (54.23)	0.17
	SCC	108 (31.76)	26 (32.50)		89 (32.01)	45 (31.69)	
	Others	30 (8.82)	13 (16.25)		23 (8.27)	20 (14.08)	
The number of tumor invading lung lobes (n, %)	1	292 (85.88)	63 (78.75)	0.11	244 (87.77)	111 (78.17)	0.01
	≥ 2	48 (14.12)	17 (21.25)		34 (12.23)	31 (21.83)	
Tumor maximum diameter, cm	Median (IR)	3.00 (3.00)	3.73 (1.75)	0.32	3.00 (2.50)	3.50 (2.50)	0.08
Neoadjuvant therapy (n, %)	No	323 (95.00)	70 (87.50)	0.01	263 (94.60)	130 (91.55)	0.23
	Yes	17 (5.00)	10 (12.50)		15 (5.40)	12 (8.45)	
ALT (U/L)	Median (IR)	19.35 (12.00)	17.70 (11.95)	0.06	19.10 (12.20)	18.80 (11.40)	0.26
AST (U/L)	Median (IR)	20.25 (9.45)	18.55 (9.65)	0.09	20.20 (9.60)	19.75 (10.10)	0.89
ALP (U/L)	Median (IR)	77.05 (27.95)	77.00 (27.10)	0.33	76.95 (28.10)	77.50 (26.60)	0.32
LDH (U/L)	Median (IR)	170.70 (46.00)	173.40 (49.50)	0.17	170.00 (48.40)	173.80 (43.00)	0.14
PDW	Median (IR)	15.75 (3.50)	15.70 (3.70)	0.78	15.60 (3.65)	15.80 (3.45)	0.41
NLR	Median (IR)	2.41 (1.58)	2.74 (1.72)	0.03	2.38 (1.53)	2.61 (1.74)	0.12
MLR	Median (IR)	0.28 (0.16)	0.31 (0.19)	0.19	0.28 (0.16)	0.30 (0.18)	0.22
PLR	Median (IR)	145.93 (71.55)	142.28 (84.79)	0.65	144.95 (67.76)	146.38 (76.68)	0.40
PNI	Median (IR)	50.50 (7.18)	50.28 (6.13)	0.11	50.65 (7.40)	49.93 (6.40)	0.03
SII	Median (IR)	527.97 (451.59)	585.78 (578.82)	0.09	525.57 (447.63)	546.16 (469.20)	0.45

IR, interquartile range; SCC, squamous cell carcinoma; ALT, alanine aminotransferase; AST, aspartate aminotransferase; ALP, alkaline phosphatase; LDH, lactate dehydrogenase; PDW, platelet distribution width; NLR, neutrophil to lymphocyte ratio; MLR, monocyte to lymphocyte ratio; PLR, platelet to lymphocyte ratio; PNI, prognostic nutritional index; SII, systemic inflammation index;

^a^P was calculated by Wilcoxon test for continuous variables and Chi-square test for categorical variables.

^b^Tumor recurrence, metastasis and death were considered as cancer progression.

### Relationships of Serum Enzymes and Blood Cytology Indices With the Prognosis of Patients With Resectable Lung Cancer

The areas under the ROC curves and cutoffs of ALT, AST, ALP, LDH, PDW, NLR, monocyte-to-lymphocyte ratio (MLR), platelet-to-lymphocyte ratio (PLR), PNI, and SII for OS and PFS are listed in **Table S3**. These indices were dichotomized with the cutoffs of the corresponding ROC curves. High levels of preoperative ALT, ALP, LDH, NLR, MLR, and SII presented significant associations with OS of patients with resectable lung cancer in univariate analyses. After adjustment for age at diagnosis, sex, smoking, history of TB, pathological stage, histological type, and neoadjuvant therapy, ALT, ALP, LDH and NLR maintained significant associations with OS with HRs (95% CI) of 3.06 (1.10-8.56), 1.99 (1.11-3.59), 1.68 (1.03-2.76), and 1.82 (1.09-3.06), respectively. ALP, LDH, and MLR present significant associations with PFS of patients with resectable lung cancer in univariate analyses. After adjustment for age at diagnosis, pathological stage, the number of tumor-invading lung lobes, histological type, and tumor maximum diameter, ALP, LDH, and PDW still presented significant associations with PFS with HRs (95% CI) of 1.63 (1.08-2.48), 1.47 (1.02-2.10), and 1.47 (1.03-2.09), respectively (**Table 2**). Considering the collinearity (**Table S4**) and the HRs of these indices, ALP and LDH were included in the integrated predictive model for OS. ALP, LDH, and PDW were incorporated into the predictive model for PFS. The survival curves of ALP and LDH for OS and PFS are shown in **Figures S1** and **S2**, respectively.

**Table 2:** The relationships of dichotomized enzymes and blood cytology indicators with prognosis of resectable lung cancer.

Enzymology, blood cytology indicators^a^	HR (95% CI) of OS		HR (95% CI) of PFS	
	Univariate	Multivariate^b^	Univariate	Multivariate^c^
ALT	3.45 (1.26-9.43)	3.06 (1.10-8.56)	2.17 (0.89-5.30)	1.96 (0.80-4.83)
AST	1.24 (0.71-2.18)	1.46 (0.82-2.57)	1.16 (0.83-1.62)	1.21 (0.86-1.69)
ALP	1.95 (1.10-3.48)	1.99 (1.11-3.59)	1.61 (1.07-2.42)	1.63 (1.08-2.48)
LDH	1.72 (1.06-2.80)	1.68 (1.03-2.76)	1.50 (1.05-2.13)	1.47 (1.02-2.10)
PDW	1.32 (0.61-2.86)	1.32 (0.60-2.87)	1.32 (0.93-1.89)	1.47 (1.03-2.09)
NLR	1.92 (1.16-3.19)	1.82 (1.09-3.06)	1.37 (0.98-1.92)	1.28 (0.91-1.80)
MLR	1.60 (1.03-2.48)	1.35 (0.86-2.11)	1.40 (1.01-1.94)	1.20 (0.86-1.68)
PLR	1.52 (0.94-2.46)	1.54 (0.94-2.52)	1.30 (0.93-1.82)	1.20 (0.86-1.69)
PNI	1.11 (0.53-2.30)	1.03 (0.49-2.17)	1.41 (0.20-10.08)	2.03 (0.28-14.64)
SII	2.15 (1.16-3.97)	1.78 (0.95-3.34)	1.47 (0.97-2.23)	1.34 (0.87-2.06)

HR, hazard ratio; CI, confidence interval; OS, overall survival; PFS, progression-free survival; ALT, alanine aminotransferase; AST, aspartate aminotransferase; ALP, alkaline phosphatase; LDH, lactate dehydrogenase; PDW, platelet distribution width; NLR, neutrophil to lymphocyte ratio; MLR, monocyte to lymphocyte ratio; PLR, platelet to lymphocyte ratio; PNI, prognostic nutritional index; SII, systemic inflammation index.

^a^Enzymes and blood cytology indicators were divided into two groups by cut-offs from the corresponding ROC curves.

^b^Multivariate Cox stepwise regression was adjusted for age at diagnosis, sex, smoking, history of tuberculosis, pathological stage, histological type and neoadjuvant therapy.

^c^Multivariate Cox stepwise regression was adjusted for age at diagnosis, pathological stage, the number of tumor invading lung lobes, histological type and tumor maximum diameter.

### Prognostic Models for Patients With Lung Cancer

**Figure 1:**
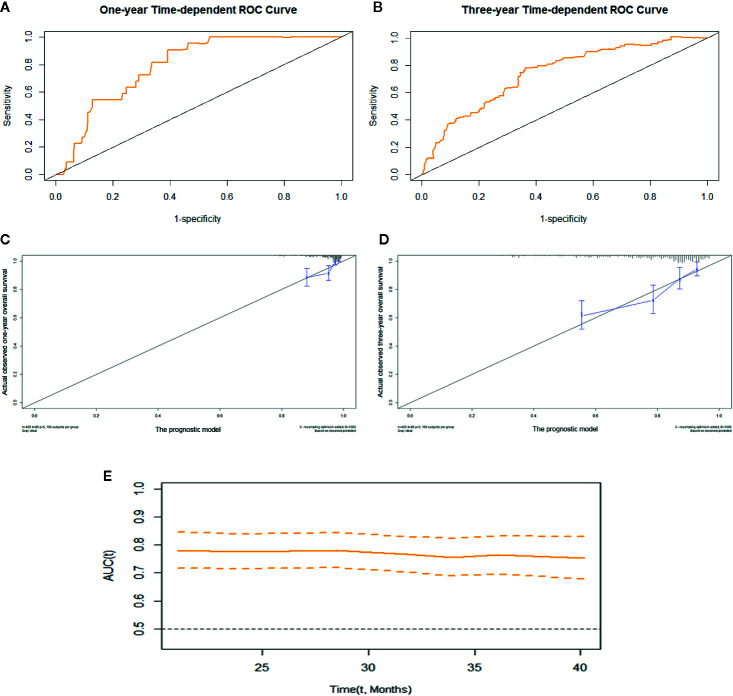
The prognostic model of OS of resectable lung cancer. The prognostic model of OS based on age, history of TB, pathological stage, ALP, and LDH. **(A)** One-year time-dependent ROC curve of the model; **(B)** 3-year time-dependent ROC curve of the model; **(C)** 1-year calibration curve of the model; **(D)** 3-year calibration curve of the model; **(E)** Time-AUC curve of the model. ALP, alkaline phosphatase; LDH, lactate dehydrogenase.

**Figure 2:**
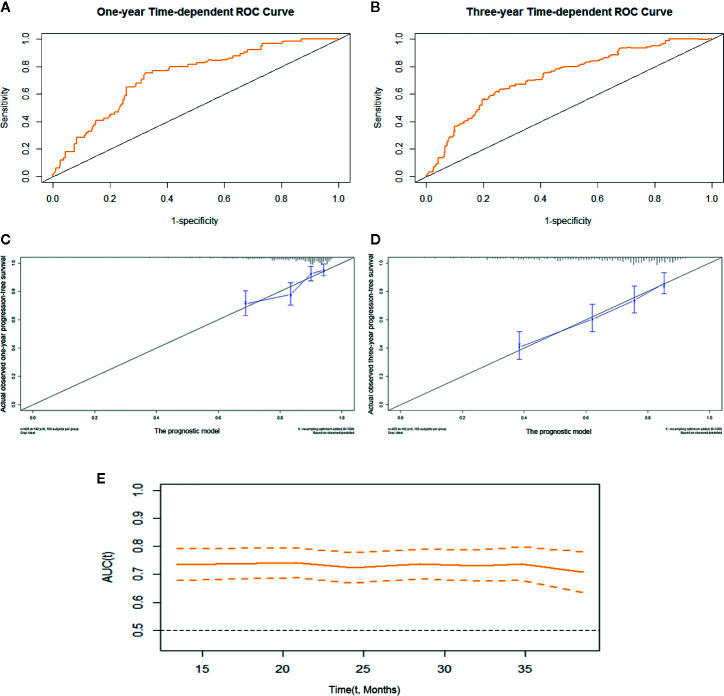
The prognostic model of PFS of resectable lung cancer. The prognostic model of PFS based on age, pathological stage, histological type, ALP, LDH, and PDW. **(A)** One-year time-dependent ROC curve of the model; **(B)** 3-year time-dependent ROC curve of the model; **(C)** 1-year calibration curve of the model; **(D)** 3-year calibration curve of the model; **(E)** Time-AUC curve of the model. ALP, alkaline phosphatase; LDH, lactate dehydrogenase; PDW, platelet distribution width.

We constructed prognostic prediction models based on serum enzymes, blood cytology indices, and clinicopathological features. The prognostic model for OS was constructed by age, history of TB, pathological stage, ALP, and LDH. The prognostic model for PFS was constructed by age, pathological stage, type of histology, ALP, LDH, and PDW. The C-indices for the prognostic models for OS and PFS were 0.74 (95% CI, 0.69-0.79) and 0.71 (95% CI, 0.66-0.75), respectively. One- and 3-year time-dependent ROC curves were generated to present the performance of the two models (**Figures 1A, B**; **2A, B**). The calibration curves also show good agreement between prediction and observation in the 1- and 3-year survival probabilities of OS and PFS with Brier scores of 0.03 and 0.08 for OS and 0.06 and 0.12 for PFS, respectively (**Figures 1C, D**; **2C, D**), and the AUCs (95% CIs) of the two models were stable over time (**Figure 1E**, **2E**).

We further performed a sensitivity analysis excluding patients with advanced lung cancer. The results show that the prognostic model based on ALP, LDH, age, history of TB, and pathological stage maintained good performance for OS prediction [C-index: 0.75 (95% CI, 0.70-0.81) and Brier score: 0.10], and the prognostic model based on ALP, LDH, PDW, age, pathological stage, and histological type maintained good performance for PFS prediction [C-index: 0.71 (95% CI, 0.67-0.76), and Brier score: 0.15]. Moreover, after adjustment for covariates, high levels of preoperative ALP and LDH maintained significant associations with OS with HRs (95% CIs) of 1.84 (1.01-3.37) and 1.99 (1.17-3.37), respectively. After adjustment for covariates, high levels of preoperative ALP, LDH, and PDW maintained significant associations with PFS, with HRs (95% CIs) of 1.56 (1.01-2.40), 1.57 (1.07-2.29), and 1.54 (1.06-2.25), respectively.

**Figure 3:**
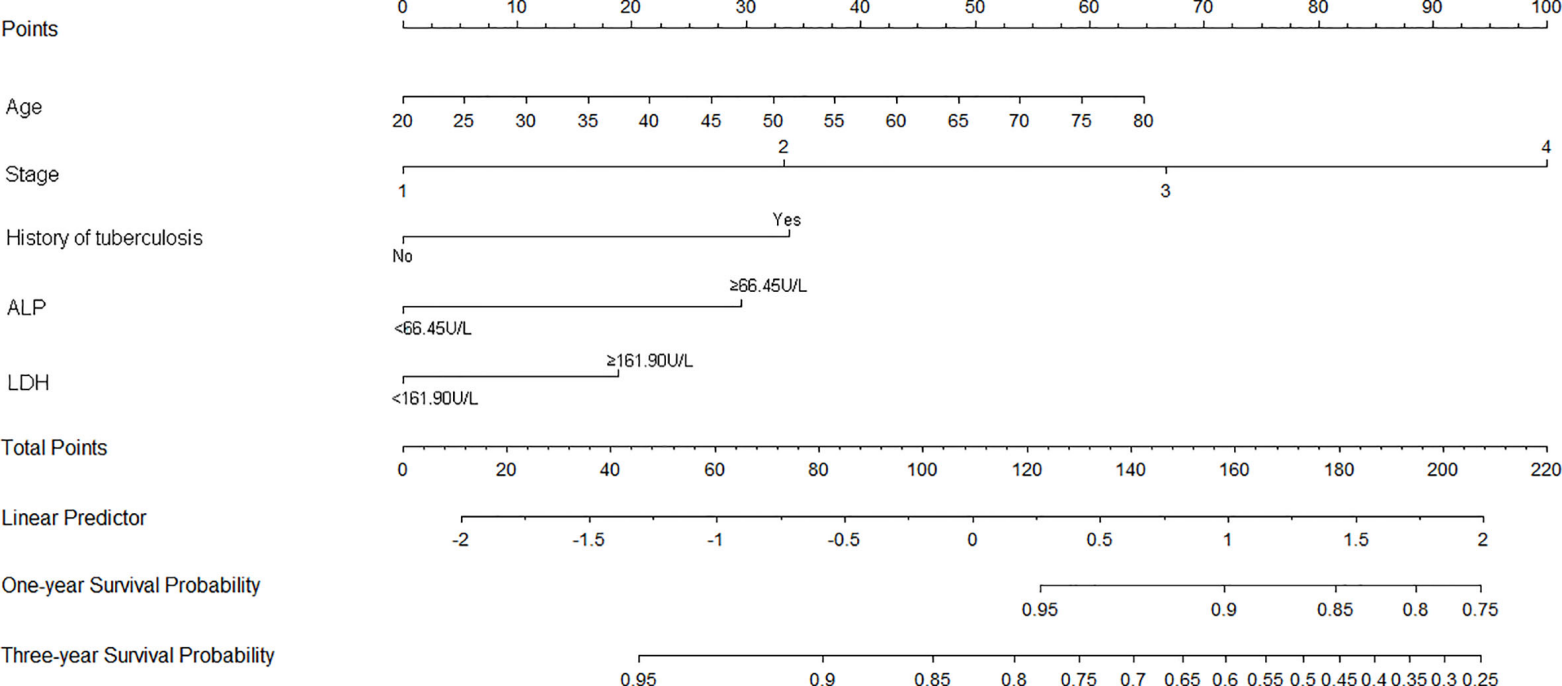
Nomogram of prognostic model for OS of resectable lung cancer. ALP, alkaline phosphatase; LDH, lactate dehydrogenase.

**Figure 4:**
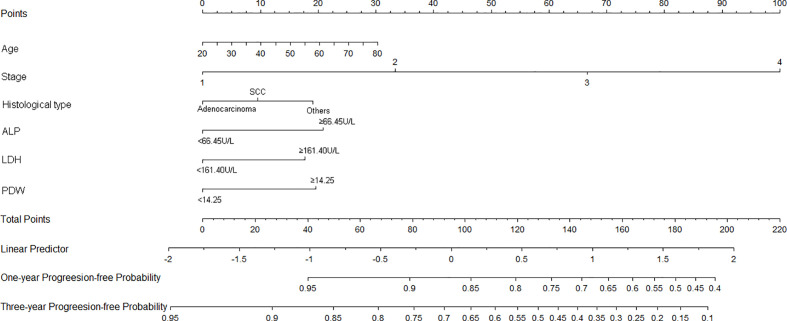
Nomogram of prognostic model for PFS of resectable lung cancer. ALP, alkaline phosphatase; LDH, lactate dehydrogenase; PDW, platelet distribution width.

Nomograms of OS and PFS were plotted based on the models as shown in **Figures 3** and **4**, respectively. The 1- and 3-year death and cancer progression risk of individual patients could be calculated by summarizing the scores of each variable point through the corresponding nomograms.

## Discussion

In general, tumor, host, and treatment are associated with cancer prognosis (16, 17). The pathological TNM stage is used for evaluating the prognosis of lung cancer in clinical practice (18). In addition to pathological TNM stage, histological type also plays a critical role in cancer progression and survival. Preexisting pulmonary history of TB is an independent risk factor for lung cancer incidence and mortality (19). We also find that the subtype of lung cancer and TB are associated with death of lung cancer. Moreover, several studies show that routine serum enzymes and blood cytology indices, such as ALP, MLR, SII, and PNI, are prognostic indices of lung cancer (20–23).

Zhang et al. (23) suggest that a high level of preoperative serum ALP (≥140 U/L) is a significant prognostic factor for bone metastasis in patients with lung cancer. Consistently, the current study reveals the adverse effect of preoperative serum ALP on tumor progression and death of patients with resectable lung cancer. Hung et al. (24) show that cancer cells contain a high ALPase activity in the nucleus, which could promote cancer cell proliferation. Therefore, it might be biologically plausible to observe high levels of ALP in patients with cancer. Deng et al. (25) suggest that higher pretreatment LDH concentration is associated with poor OS in patients with lung cancer. The current study shows that preoperative high serum LDH levels are independent risk factors for OS and PFS. Lee et al. (26) also find that, compared with the low metastatic score group, the patients in the high metastatic score group had significantly higher levels of serum LDH, which might be a mechanism for the link between a high level of LDH and poor OS and PFS in lung patients with cancer. A retrospective study of non-small cell lung cancer shows that a high preoperative level of PDW is a poor prognostic factor for OS in patients with lung cancer (27), and this study shows that a high level of PDW is a poor prognostic factor for PFS. The biological mechanism of the association of PDW and cancer progression is still under discussion.

The AUCs of previous prognostic models for OS of lung patients with cancer based on a prospective cohort study ranged from 0.62 to 0.71, which were developed based on performance status, age, sex, tumor (T) and node (N) stage, tumor volume, total radiotherapy dose, and chemotherapy timing (28). A prognostic model constructed by age, T stage, lymph node status, and grade presents C-indices of 0.68 (95% CI: 0.63–0.73), 0.66 (95% CI: 0.61–0.71), and 0.68 (95% CI: 0.63–0.72) for disease-free survival, cancer-specific survival, and OS of squamous cell lung cancer, respectively (29). A study based on Surveillance, Epidemiology, and End Results constructed a nomogram based on age, sex, the total number of sites, histological types, grade, tumor size, and treatment and presented a C-index of 0.72 for OS (30). In the current study, prognostic models of patients with lung cancer were constructed by combining serum enzymes, blood cytology indices, and clinicopathological characteristics. The model based on age, history of TB, pathological stage, ALP, and LDH had excellent performance for OS of patients with lung cancer with a C-index of 0.74 (95% CI: 0.69-0.79). The model based on age, pathological stage, histological type, ALP, LDH, and PDW had remarkable predictive performance for PFS with a C-index of 0.71 (95% CI: 0.66-0.75). The predictive models are plotted as nomograms, which can be used to individually predict death and cancer progression in patients with lung cancer. In addition to age, history of TB, pathological stage, and histological type, the models innovatively include ALP, LDH, and PDW, which are routine indicators for patients. Collinearity is excluded in the screening of serum enzymes and blood cytology indices, which not only ensures the effectiveness of the models, but also makes them more convenient in clinical practice.

The current study has limitations. Our study is a retrospective study that includes patients who underwent an operation. Part of the outcome information was collected by making follow-up calls, which could generate loss of follow-up. In addition, the sample size in this study was limited. The prognostic models of lung cancer were not validated, and the generalization performance is unknown. The mechanism of the associations between serum enzymes, blood cytology indices, and prognosis of patients with lung cancer is unclear. Nevertheless, this study developed prognostic models based on routine, easily accessible indices in clinical practice, and plotted them as nomograms for visual prediction.

In conclusion, ALP and LDH are prognostic predictors of OS, and ALP, LDH, and PDW are prognostic indices of PFS in patients with lung cancer. Models based on serum enzymes, blood cytology indices, and clinicopathological characteristics are good for prognosis prediction of lung cancer and might be used as convenient tools for individualized evaluation of cancer progression and death.

## Data Availability Statement

The raw data supporting the conclusions of this article will be made available by the authors, without undue reservation.

## Ethics Statement

Written informed consent was obtained from all patients before this study. The study was approved by the ethical committee of Hubei Cancer Hospital.

## Author Contributions

YYL: Writing—review and editing, funding acquisition. XYL: Software, writing—original draft. ZCY: Data curation, methodology. PL: Data curation, investigation. YFM: Data curation, methodology. JDK: Data collections, data analysis. BL: Formal analysis, validation. SZW: Conceptualization, project administration. XJL: Funding acquisition, supervision. All authors contributed to the article and approved the submitted version.

## Funding

This work was financially supported by the National Nature Science Foundation of China (NSFC) (grant numbers 81572287, 81772499 and 81974088), Health commission of Hubei Province scientific research project (WJ2017M142), Natural Science Foundation of Hubei Province (No.2017CFB555), and Foundation of Chinese Society of Clinical Oncology (CSCO: Y-HS2019-39, Y-MX2016-048).

## Conflict of Interest

The authors declare that the research was conducted in the absence of any commercial or financial relationships that could be construed as a potential conflict of interest.
