# Supplementary material for: Prognostic Prediction Models Based on Clinicopathological Indices in Patients With Resectable Lung Cancer
Source: Front Oncol. 2020 Oct 29;10:571169. doi: 10.3389/fonc.2020.571169 (PMC7658583; doi:10.3389/fonc.2020.571169)
Supplement: Supplementary file 1 [file DataSheet_1.docx]

**Supplemental Methods**

**Patients and follow-up methods**

Four hundred and eighty-seven primary lung cancer patients who underwent first tumor resection at Hubei Cancer Hospital from January 2015 to June 2017 were recruited. The patients were excluded if (1) they censored within 90 days from tumor resection; (2) they had previous or concurrent malignancies; (3) they had preexisting inflammatory conditions such as active or chronic infection. Finally, 420 patients were included in this study. Written informed consent was obtained from all patients before this study. The study was approved by the ethical committee of Hubei Cancer Hospital.

We obtained outcomes by reviewing medical records and taking follow-up calls. The main outcome was OS, and the secondary outcome was PFS. The OS was defined as the interval from the date of tumor resection to the date on which patients died from any cause, lost to follow-up, or the end of the follow-up, whichever came first. The PFS was defined as the interval from the date of tumor removal to the date on which patients died, recurrence or metastasis was detected, lost to follow-up, or the end of the follow-up, whichever came first. Follow-up was carried out to the end of February 2019.

**Statistical analysis**

Continuous variables were presented as mean ± standard deviation (SD), or median and interquartile range (IR), and the student’s t test or wilcoxon test was used for comparison between groups. Categorical variables were expressed by counts and percentages, and the chi-square test was used for comparison between groups. Five joint indicators were constructed, including NLR, MLR, platelet to lymphocyte ratio (PLR), PNI and SII. Receiver operating characteristic (ROC) curves were applied to transform the continuous variables (ALT, AST, ALP, LDH, PDW, NLR, MLR, PLR, PNI and SII) into dichotomized variables by using inflexion points as cut-offs. Kaplan-Meier survival curves and log-rank test were used to compare the survival difference between groups classified by dichotomized clinicopathological indicators. Univariate and multivariate Cox proportional hazards regression were applied to detect the associations of individual clinicopathological features, and integrated predictive models based serum enzymes, blood cytology indicators and other clinicopathological characteristics with OS/PFS by calculating hazard ratios (HRs) and 95% confidence intervals (95% CIs). Prognostic efficacy of the predictive models was estimated by Harrell's concordance index (C-index) and Brier score. Time-dependent ROC curves and calibration curves were plotted to visualize the performance of the models[30]. Nomograms of the predictive models were plotted for individualized evaluation of OS and PFS.

All statistical tests were two-sided, and *P<0.05* was considered as statistically significant. Time-dependent receiver operating characteristic curve, calibration curve and nomogram were performed using packages “survivalROC”, “timeROC”, “pec” and “regplot” of R 3.6.0 (The R Foundation for Statistical Computing, Vienna, Austria), respectively. Other statistical analyses were performed using the SAS Statistics software 9.4 (SAS Institute Inc, Cary, North Carolina, USA).
