# Supplementary material for: Prognostic Prediction Models Based on Clinicopathological Indices in Patients With Resectable Lung Cancer
Source: Front Oncol. 2020 Oct 29;10:571169. doi: 10.3389/fonc.2020.571169 (PMC7658583; doi:10.3389/fonc.2020.571169)
Supplement: Supplementary file 2 [file Table_1.docx]

**Table S1. The relationships between clinicopathological characteristics with OS**

| **Variables** | | **Univariate Analysis** | |  | **Multivariate Analysis** | |
| --- | --- | --- | --- | --- | --- | --- |
|  |  | **HR ( 95% CI)** | ***P*** |  | **HR ( 95% CI)** | ***P*** |
| Age at diagnosis, y | <65 | 1.00 |  |  | 1.00 |  |
|  | ≥65 | 1.29 (0.80-2.07) | 0.29 |  | 1.32 (0.81-2.13) | 0.27 |
| Sex | Male | 1.00 |  |  | 1.00 |  |
|  | Female | 0.56 (0.33-0.94) | 0.03 |  | 0.82 (0.35-1.94) | 0.65 |
| Smoking | No | 1.00 |  |  | 1.00 |  |
|  | Yes | 1.81 (1.13-2.90) | 0.01 |  | 1.45 (0.66-3.17) | 0.35 |
| Body mass index, kg/m^2^ | <24 | 1.00 |  |  |  |  |
|  | ≥24 | 0.98 (0.59-1.63) | 0.95 |  |  |  |
| History of tuberculosis | No | 1.00 |  |  | 1.00 |  |
|  | Yes | 2.07 (1.03-4.15) | 0.04 |  | 2.18 (1.07-4.44) | 0.03 |
| Pathological stage | Each stage up | 1.97 (1.59-2.44) | <0.0001 |  | 1.90 (1.54-2.43) | <0.0001 |
| The number of tumor invading lung lobes | <2 | 1.00 |  |  |  |  |
|  | ≥2 | 1.55 (0.91-2.65) | 0.11 |  |  |  |
| Histological type | Adenocarcinoma | 1.00 |  |  | 1.00 |  |
|  | SCC | 1.19 (0.73-1.94) | 0.49 |  | 0.83 (0.48-1.43) | 0.50 |
|  | Others | 2.06 (1.10-3.85) | 0.02 |  | 1.89 (0.99-3.62) | 0.05 |
| Tumor maximum diameter, cm | <3 | 1.00 |  |  |  |  |
|  | ≥3 | 1.40 (0.87-2.24) | 0.17 |  |  |  |
| Neoadjuvant therapy | No | 1.00 |  |  | 1.00 |  |
|  | Yes | 2.65 (1.37-5.15) | 0.0004 |  | 2.00 (0.98-4.06) | 0.06 |

HR, hazard ratio; CI, confidence interval; SCC, squamous cell carcinoma.

**Table S2. The relationships between clinicopathological characteristics with PFS**

| **Variables** | | **Univariate Analysis** | |  | **Multivariate Analysis** | |
| --- | --- | --- | --- | --- | --- | --- |
|  |  | **HR ( 95% CI)** | ***P*** |  | **HR ( 95% CI)** | ***P*** |
| Age at diagnosis, y | <65 | 1.00 |  |  | 1.00 |  |
|  | ≥65 | 1.07 (0.74-1.55) | 0.71 |  | 1.17 (0.80-1.70) | 0.43 |
| Sex | Male | 1.00 |  |  |  |  |
|  | Female | 0.73 (0.50-1.05) | 0.09 |  |  |  |
| Smoking | No | 1.00 |  |  |  |  |
|  | Yes | 1.36 (0.97-1.91) | 0.08 |  |  |  |
| Body mass index, kg/m^2^ | <24 | 1.00 |  |  |  |  |
|  | ≥24 | 1.02 (0.70-1.50) | 0.94 |  |  |  |
| History of tuberculosis | No | 1.00 |  |  |  |  |
|  | Yes | 1.49 (0.82-2.69) | 0.19 |  |  |  |
| Pathological stage | Each stage up | 1.83 (1.56-2.15) | <0.0001 |  | 1.81 (1.53-2.15) | <0.0001 |
| The number of tumor invading lung lobes | <2 | 1.00 |  |  | 1.00 |  |
|  | ≥2 | 1.65 (1.11-2.46) | 0.01 |  | 1.21 (0.79-1.847) | 0.38 |
| Histological type | Adenocarcinoma | 1.00 |  |  | 1.00 |  |
|  | SCC | 1.05 (0.73-1.52) | 0.79 |  | 0.92 (0.63-1.35) | 0.67 |
|  | Others | 1.74 (1.06-2.85) | 0.02 |  | 1.78 (1.08-2.95) | 0.02 |
| Tumor maximum diameter, cm | <3 | 1.00 |  |  | 1.00 |  |
|  | ≥3 | 1.49 (1.04-2.13) | 0.03 |  | 1.15 (0.79-1.68) | 0.48 |
| Neoadjuvant therapy | No | 1.00 |  |  |  |  |
|  | Yes | 1.57 (0.87-2.84) | 0.13 |  |  |  |

HR, hazard ratio; CI, confidence interval; SCC, squamous cell carcinoma.

**Table S3. The calculations, cut-off points and AUCs of enzymes and blood cytology indicators**

| **Indicators** | **Calculation** | **Overall survival** | |  | **Progression-free survival** | |
| --- | --- | --- | --- | --- | --- | --- |
|  |  | **Cut-off** | **AUC (95% CI)** |  | **Cut-off** | **AUC (95% CI)** |
| ALT, (U/L) |  | 83.45 | 0.43 (0.36-0.51) |  | 78.00 | 0.47 (0.41-0.53) |
| AST, (U/L) |  | 28.55 | 0.44 (0.36-0.56) |  | 23.35 | 0.49 (0.43-0.53) |
| ALP, (U/L) |  | 66.45 | 0.54 (0.47-0.61) |  | 66.45 | 0.53 (0.47-0.59) |
| LDH, (U/L) |  | 161.90 | 0.55 (0.48-0.62) |  | 161.40 | 0.54 (0.49-0.60) |
| PDW |  | 16.75 | 0.49 (0.42-0.56) |  | 14.25 | 0.53 (0.47-0.58) |
| NLR | Neutrophil count / lymphocyte count | 2.09 | 0.58 (0.51-0.64) |  | 2.40 | 0.55 (0.49-0.61) |
| MLR | Monocyte count / lymphocyte count | 0.31 | 0.54 (0.47-0.61) |  | 0.31 | 0.54 (0.48-0.59) |
| PLR | Platelet count / lymphocyte count | 186.90 | 0.52 (0.45-0.59) |  | 165.80 | 0.53 (0.47-0.59) |
| PNI | Albumin + lymphocyte count × 5 | 44.15 | 0.44 (0.38-0.51) |  | 33.95 | 0.43 (0.38-0.49) |
| SII | Platelet count × neutrophil count / lymphocyte count | 364.33 | 0.56 (0.49-0.63) |  | 1047.39 | 0.53 (0.47-0.59) |

AUC, area under the receiver operating characteristic curve; CI, confidence interval; ALT, alanine aminotransferase; AST, aspartate aminotransferase; ALP, alkaline phosphatase; LDH, lactate dehydrogenase; PDW, platelet distribution width; NLR, neutrophil to lymphocyte ratio; MLR, monocyte to lymphocyte ratio; PLR, platelet to lymphocyte ratio; PNI, prognostic nutritional index; SII, systemic inflammation index.

**Table S4. The linear correlation analysis of enzymes and blood cytology indicators**

|  | **Pearson correlation coefficient (*P ^a^*)** | | | | | | | | | |
| --- | --- | --- | --- | --- | --- | --- | --- | --- | --- | --- |
|  | **ALT** | **AST** | **ALP** | **LDH** | **PDW** | **NLR** | **MLR** | **PLR** | **PNI** | **SII** |
| ALT | 1.00 |  |  |  |  |  |  |  |  |  |
| AST | 0.67 (<0.0001) | 1.00 |  |  |  |  |  |  |  |  |
| ALP | 0.16 (0.001) | 0.14 (0.005) | 1.00 |  |  |  |  |  |  |  |
| LDH | 0.11 (0.02) | 0.18 (0.0002) | 0.06 (0.19) | 1.00 |  |  |  |  |  |  |
| PDW | 0.12 (0.01) | 0.11 (0.02) | 0.04 (0.44) | 0.08 (0.09) | 1.00 |  |  |  |  |  |
| NLR | 0.04 (0.44) | -0.04 (0.37) | 0.22 (<0.0001) | 0.07 (0.17) | 0.03 (0.51) | 1.00 |  |  |  |  |
| MLR | 0.04 (0.33) | -0.005 (0.92) | 0.11 (0.03) | 0.006 (0.89) | -0.11 (0.02) | 0.52 (<0.0001) | 1.00 |  |  |  |
| PLR | -0.08 (0.10) | -0.11 (0.03) | 0.22 (<0.0001) | 0.05 (0.32) | -0.18 (0.0002) | 0.55 (<0.0001) | 0.32 (<0.0001) | 1.00 |  |  |
| PNI | 0.12 (0.01) | 0.15 (0.002) | -0.04 (0.43) | 0.17 (0.0004) | 0.07 (0.15) | -0.30 (<0.0001) | -0.30 (<0.0001) | -0.37 (<0.0001) | 1.00 |  |
| SII | 0.01 (0.80) | -0.09 (0.06) | 0.27 (<0.0001) | 0.04 (0.44) | -0.09 (0.06) | 0.89 (<0.0001) | 0.43 (<0.0001) | 0.74 (<0.0001) | -0.30 (<0.0001) | 1.00 |

ALT, alanine aminotransferase; AST, aspartate aminotransferase; ALP, alkaline phosphatase; LDH, lactate dehydrogenase; PDW, platelet distribution width; NLR, neutrophil to lymphocyte ratio; MLR, monocyte to lymphocyte ratio; PLR, platelet to lymphocyte ratio; PNI, prognostic nutritional index; SII, systemic inflammation index;

^a^ *P < 0.05* means that linear correlation test was statistically significant.

Figure S1: Kaplan-Meier curves of overall survival of resectable lung cancer by (A) ALP, and (B) LDH.

Figure S2: Kaplan-Meier curves of progression-free survival of resectable lung cancer by (A) ALP, and (B) LDH.
