# Supplementary figures and images for: Prognostic Prediction Models Based on Clinicopathological Indices in Patients With Resectable Lung Cancer
Source: Front Oncol. 2020 Oct 29;10:571169. doi: 10.3389/fonc.2020.571169 (PMC7658583; doi:10.3389/fonc.2020.571169)

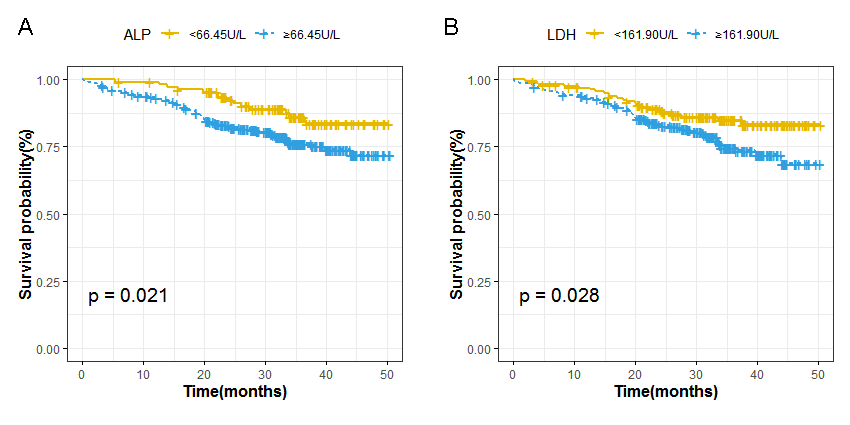

Supplement: Supplementary file 3 [file Image_1.tiff]

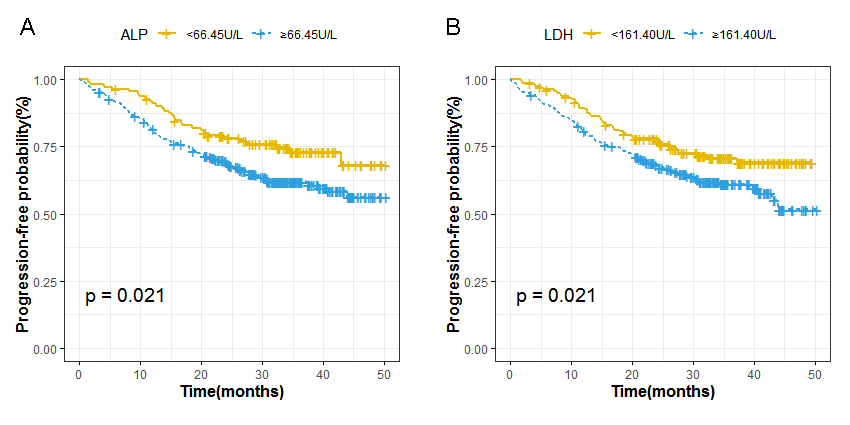

Supplement: Supplementary file 4 [file Image_2.tiff]
